# Inter-Rater and Test-Retest Reliability of an Innovative Evaluation Tool: CrossFit Functional Assessment Battery of Tests for the Shoulder Joint

**DOI:** 10.7759/cureus.53267

**Published:** 2024-01-30

**Authors:** Akrivi Bakaraki, Dionisis Parmaxizoglou, Panagiotis Gkrilias, Maria Tsekoura, Konstantinos Fousekis, Sofia Xergia, Charalampos Matzaroglou, Elias Tsepis

**Affiliations:** 1 Laboratory of Therapeutic Exercise and Sports Rehabilitation, Department of Physiotherapy, School of Health Rehabilitation Sciences, University of Patras, Patras, GRC; 2 Biomechanics Laboratory, Department of Physiotherapy, School of Health Sciences, University of Peloponnese, Sparta, GRC

**Keywords:** prevention, asymmetry, test-battery, functional assessment, screening, crossfit, injury risk, shoulder injuries

## Abstract

Background and objectives

This study aims to introduce an innovative functional assessment tool designed for CrossFit athletes, to identify a high risk of injury at the shoulder joint. Additionally, the study seeks to examine both inter-rater reliability, which was tested in 40 CrossFit participants, and test-retest reliability, which was assessed in twenty subjects.

Methodology

CrossFit Functional Assessment Battery for the Shoulder Joint (CrossFit FABS) is a newly created instrument presented for the first time. The evaluation of the performance of its six items aimed to reveal deficits that could contribute to incidents of shoulder injuries. For this purpose, 40 healthy CrossFit participants were concurrently but independently examined by two raters, and twenty healthy adults active in sports were assessed by the main investigator at two different time points. Cohen’s kappa coefficient was used to analyze categorical data with an ordinal structure.

Results

Inter-rater reliability ranged from 0.824 to 1 (*P *= 0.000) and test-retest reliability was 0.661 to 0.906 (*P *< 0.001) for each test of CrossFit FABS. A strong to almost perfect correlation was demonstrated for all the variables between the two examiners. Moderate to almost perfect correlation was shown through test-retest procedures.

Conclusions

The proposed test battery was established as a reliable tool for evaluating performance routines that represent high injury-risk elements for the shoulder joint in CrossFit athletes.

## Introduction

CrossFit is prescribed as "a constantly varied, high-intensity, functional movement" [1]. It is a highly motivational way of training [2] based on preparation for random physical challenges [1]. Each day's workout consists of a wide variety of different exercise modalities, such as calisthenics, gymnastics, metabolic conditioning, and weightlifting, which includes both Olympic and powerlifting movements. Exercises are performed at high intensity, with minimal or no rest periods between sets, aiming for the maximum number of repetitions in a time cap (AMRAP) or as quickly as possible for the best time. Workout of the day (WOD) is conducted individually or in a team of two or more persons [1,3]. Its popularity has increased in recent years, with more than 300,000 athletes competing worldwide in 2023 [4] and many more practitioners of a lower level. Some of the special characteristics of this training model are the briefness of the workouts and the scaling option for every single exercise to be performed safely and effectively for each participant [5]. Except for scaling individual movements, alterations are made to volume, including total repetitions, load, and range of motion. These adjustments aim to maintain the programmed stimuli customized for each athlete's class or condition [1,5].

While it is observed that safety is a priority within the framework of this sport, concerns about the potential riskiness of CrossFit have been raised nonetheless. Accordingly, in recent years, researchers worldwide have started to investigate the prevalence of injuries in CrossFit [6-14]. The majority of studies were retrospective and collected their data through electronic self-reported questionnaires. To the researchers’ knowledge, only three prospective studies in the literature followed a cohort sample for eight to 12 weeks to identify injury incidents during sports participation [12-14]. Two recent reviews of the current literature - one brief [15] and one systematic, including a larger proportion of included studies compared to other reviews [16] - indicated injury incidence rates of 0.27-3.3 per 1,000 training hours and 0.2-18.9, respectively. It is a consensus that the shoulder, spine, and knee are hierarchically the most injured areas in CrossFit, as indicated in a preliminary study in Greece [17], with injury incidents ranging between 12.8% and 73.5% [15] or a mean of 35.3% [16] of total participants. Previous injuries, longer training experience, and participation in competitions are associated with a higher risk of injury [15,16]. Regarding shoulder injuries, an injury incidence of 23.5% is reported by the sole retrospective study in the literature, which specifically investigated shoulder injuries in CrossFit [18]. Most of the injuries were either an aggravation of a previous injury or resulted from improper technique, both at the same percentage of 33.3% each [18]. The most common causes were shown to be gymnastics and weightlifting movements in equal measure, with an emphasis on overhead exercises such as snatching and pressing variations [18].

Both gymnastics and weightlifting demand sufficient kinetic control at an excessive range of motion of the shoulders and high forces production distally while maintaining stability centrally [19,20]. A first step in developing preventive techniques, both in research and practice, could involve identifying participants who may face a higher risk of injury through pre-participation or pre-season assessments in the gym. Principles of strength, flexibility, and endurance are validated as playing a critical role in injury incidence rates. Similarly, functional competency is identified as a crucial factor as well [21]. CrossFit is characterized by a complexity of movements requiring explosive power production in terms of stability. The multifaceted nature of the sport necessitates meticulous screening, which should comprise a variety of tests to assess shoulder function in a holistic manner [1,19-21]. In recent years, there has been a recommendation to design and use test batteries instead of isolated tests in similar studies concerning joint screenings for injury susceptibility [22]. The required evaluation tools should be familiar to coaches and athletes and should be based on scientific evidence. Surprisingly, there is no specially designed instrument for the sport in the literature. However, in practice, coaches use exercises to scale athletes, and athletes utilize these as accessory exercises to test and enhance their performance. In addition, there are no specialized evaluation tools to identify shoulder functional deficits. Functional parameters have not been evaluated among CrossFitters except for Functional Movement Screen (FMS) [23]. Although this screening tool is particularly useful in testing qualitative parameters of movement performance, its predictability of injury incidence remains disputable [24]. Some researchers have created their batteries to investigate specific athletic populations [25]. Consequently, a reliable functional assessment tool needed to be CrossFit-specific and easily applicable to practice at a low cost to facilitate future research on the topic.

This study aims to introduce a novel, sport-specific functional evaluation battery for CrossFit and evaluate its inter-rater and intra-rater reliability. The results are anticipated to contribute to a wider investigation of the risks associated with shoulder injuries in CrossFit.

## Materials and methods

A sport-specific functional assessment tool was developed by researchers in the primary stage of investigation. It included four elements related to basic characteristics of CrossFit performance: mobility-core stability-kinetic control, Olympic weightlifting technique, shoulder, and balance-strength. Twenty tests, five for each category, comprised that extensive tool aimed at identifying deficits and asymmetries capable of leading to various future injuries. A pilot study was conducted to assess the applicability and qualitative reliability of that screening tool among three different health professionals, a physiotherapist researcher and a six-year experienced CrossFit athlete who was the main researcher, an Olympic weightlifting and CrossFit coach, and a physiotherapist researcher with no experience in CrossFit. A comprehensive briefing, informative instructions, and a photo guide were provided by the main researcher to all examiners. That pilot study took place in a CrossFit-affiliated gym using a sample of 10 athletes. Preliminary outcomes of the study resulted in the isolation of the most reliable items related to shoulder injury risks. For instance, the technique section was excluded from the final evaluation tool despite its significance, due to high divergence among examiners' scores [26]. Contrary to the coach, researchers were not capable of identifying critical mistakes in Olympic weightlifting exercises’ performance. Subsequently, the finalized shoulder evaluation battery, called CrossFit FABS (Functional Assessment Battery for the Shoulder Joint), was tested for reliability via two separate sample groups: (1) 40 healthy CrossFit participants for the inter-rater examination and (2) 20 healthy collegian athletes for the test-retest examination. Test-retest group participants were examined by the main researcher for the first time and after one week for the second time. Specific instructions were given to the participants about the week between the two measurements. They were strongly advised not to change their weekly training routine until they were reassessed. Participants who had undergone surgery, experienced an injury, or had a medical condition within the last three months were excluded from the study. All participants signed informed consent, and their demographic data were reported in standardized forms. A general health and medical history form, along with a specific history of shoulder joint injuries, was completed. Training characteristics, including volume and experience, were recorded. The test-retest procedure was conducted at the Therapeutic Exercise Laboratory in the Physiotherapy Department of the University of Patras. The investigation of inter-rater reliability took place in CrossFit-affiliated gyms in four different cities in Greece. The second investigator who took part in the measurements had previously undergone training using instructional materials similar to those described in the pilot study by the main experienced researcher. The study protocol had obtained ethical approval from the Ethics Committee of the University of Patras (ID 14279) and had been registered in the ClinicalTrials.gov PRS database (Identifiers NCT05909592).

CrossFit FABS test: scoring system

The instrument consists of six items involving functional and mobility movements: air squat, shoulder mobility, wall angel, overhead squat (OHS), kettlebell (kb) windmill, and Sots press. The required equipment is available in every gym and contains a plastic stitch used in warm-up and technique exercises, a 6-kg kb for women and an 8-kg kb for men, a women’s Olympic bar and a men’s Olympic bar, and some chalk for the kb grip. All the participants were performing the tests barefoot and shirtless. Examiners described and presented the correct execution of each movement before each test, and the participants took some time to familiarize themselves with the patterns. A written guide with specific verbal instructions was compiled by the main researcher to ensure that the same procedure would be followed, which is presented in detail later.

Air Squat

**Figure 1:**
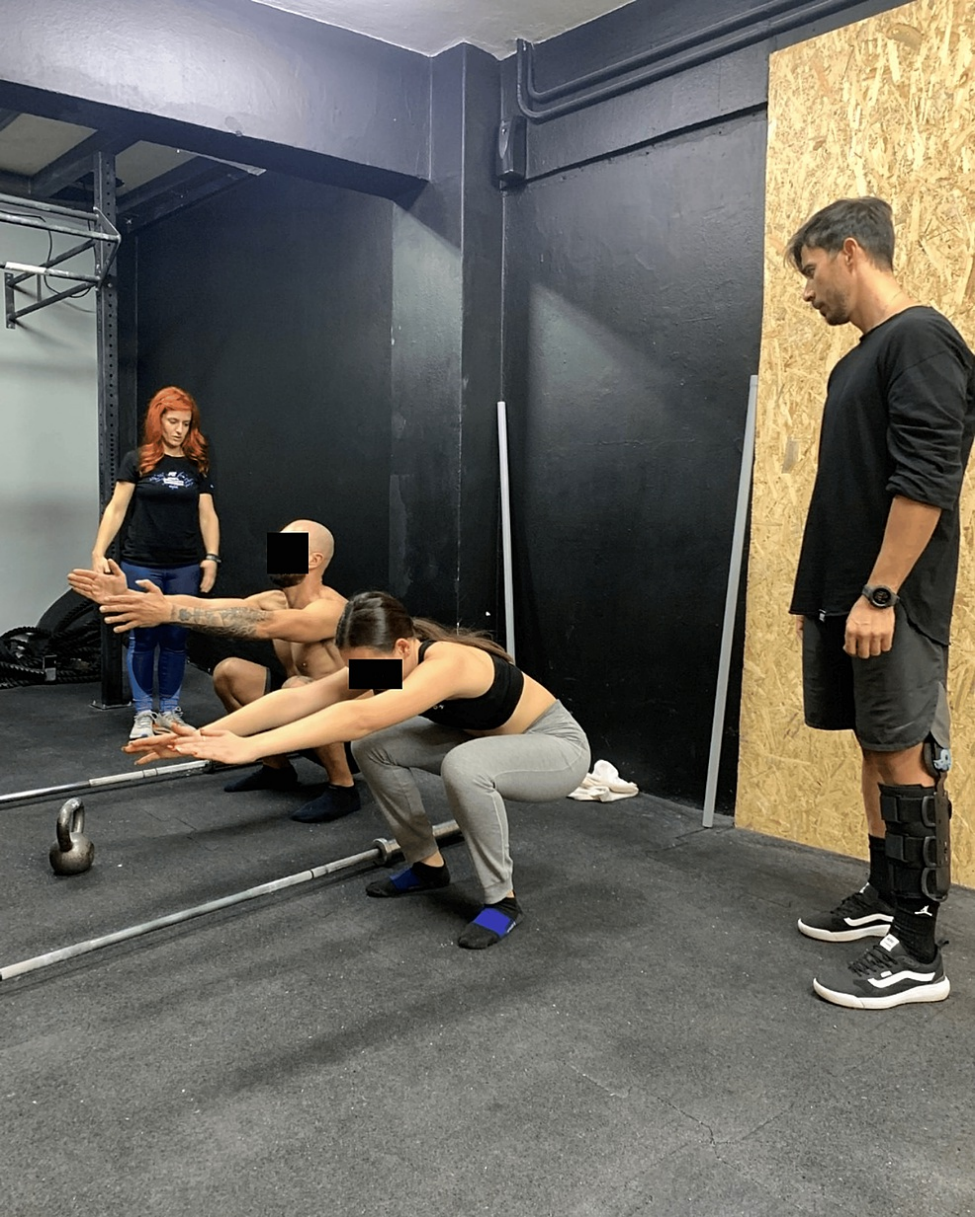
The air squat test.

Stand tall with your feet shoulder-width apart and toes pointing forward and slightly to the sides, about 15° out of the heel line [27]. Bring your arms extended forward at the level of your shoulders. Squat as deep as possible, keeping your trunk upright and your arms at shoulder level. Maintain your heels attached to the ground and do not touch your legs. Look straight forward and hold this position for three seconds (Figure 1).

Shoulder Mobility

**Figure 2:**
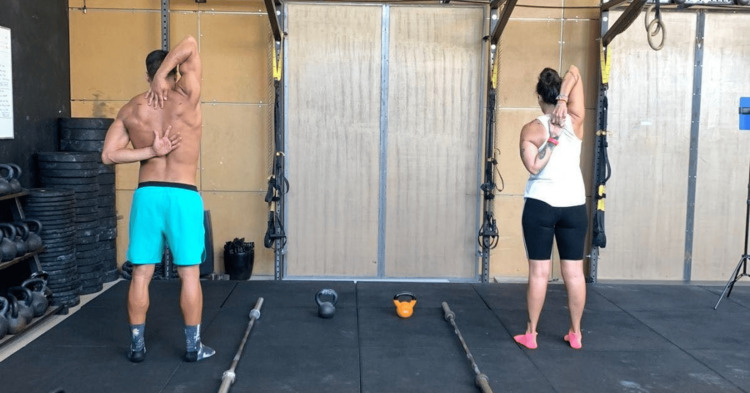
The shoulder mobility test.

Stand tall in the previous initial position and bring your right hand back to your spine [23,24]. Keep your hand open, with your palm facing out. Bring your left hand over your head and down to your back. Keep it open with your left palm facing your body. Try to clasp your fingers together as much as possible. Do not pull your arm with the other arm to go closer. Hold this position for three seconds and then do the opposite. A one-minute time interval was provided between tests (Figure 2). (The first score is for the right hand, and the second for the left. The examiner records both as independent tests.)

Wall Angel

**Figure 3:**
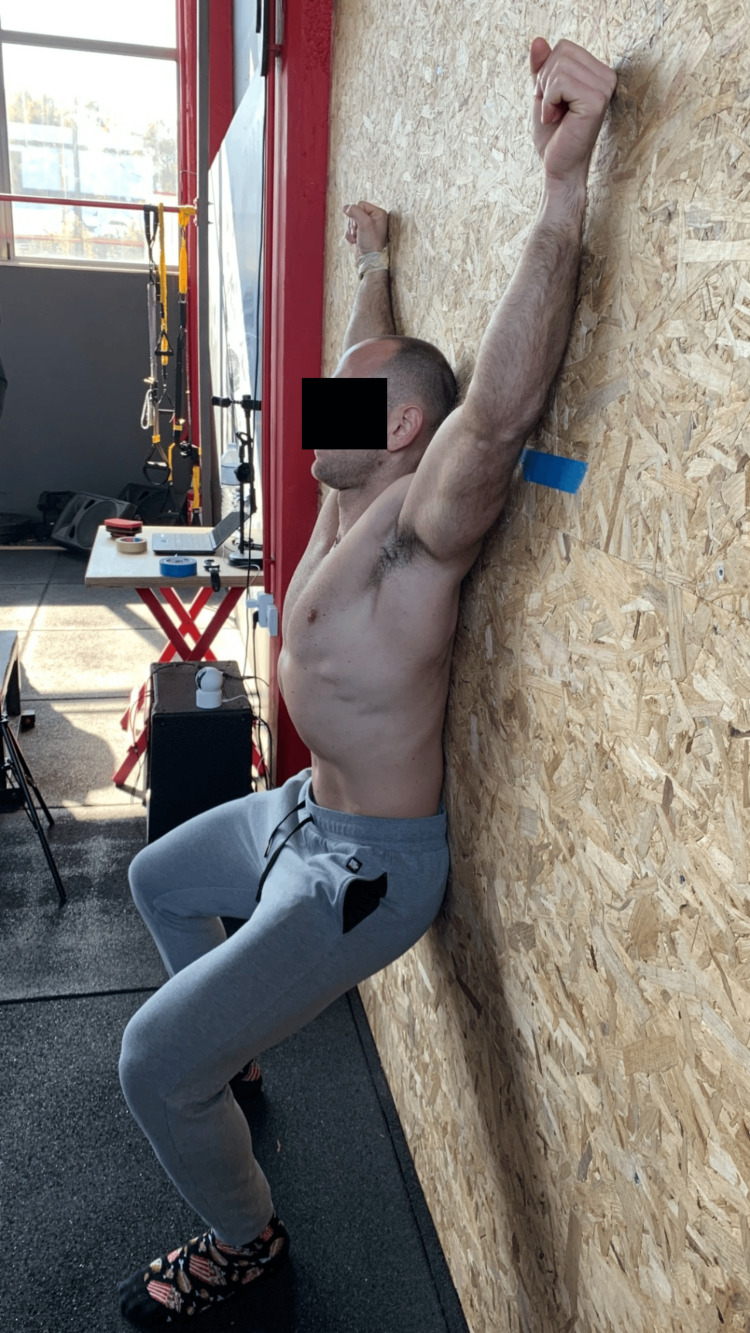
The wall angel test.

Stand tall, with your head and your back on the wall and your feet shoulder-width apart [28]. Move your feet as forward as you need to attach your lower back to the wall. (From this position, the examiner places participants’ shoulders at 90° abduction and external rotation, with elbows at 90° flexion and wrists attaching to the wall.) Do not move your wrists from this point on the wall, as if somebody is holding you there. Lower down until your elbows straighten. Try to keep your head, shoulders, elbows, and wrists in touch with the wall as you descend. Hold this position for three seconds (Figure 3).

OHS

**Figure 4:**
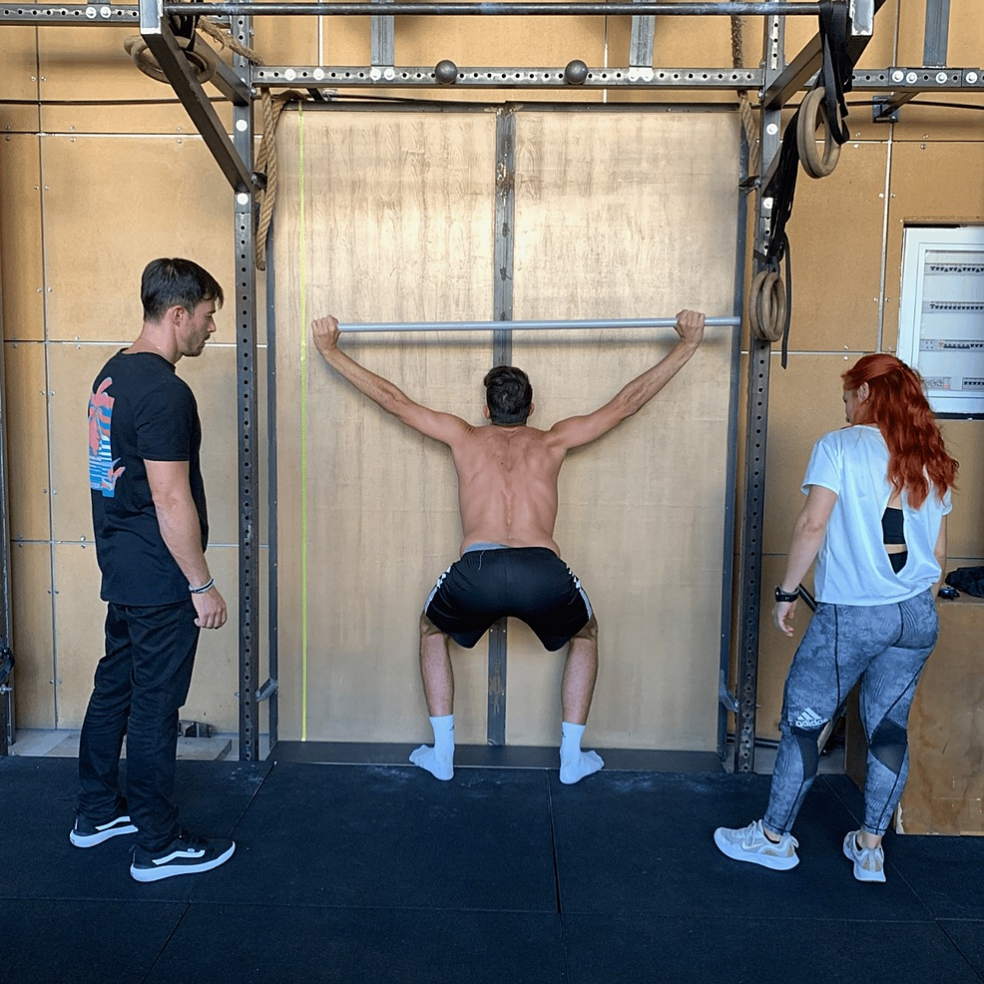
The OHS. OHS, overhead squat

Measure the distance of your fist across the wall and point your toes there [23,24]. Maintain that distance from the wall for this test. Stand tall with your feet slightly out of shoulder width. Toes should point forward and slightly to the sides, about 15° outward from the heel line. Grasp the stick in an overhead position with the handle of the snatch. To find this out, grip the bar at a width that places it in the crease of your hip when holding it at arm’s length. (Examiner at this point ensures that the bar contacts soft tissue between the anterior superior iliac spines and the pubic bone for the proper handle. Athletes with mobility or stability deficits usually prefer to make their grip wider, so the examiner corrects this during the initial stage.) Now, descend into an OHS as deeply as you can. Keep your trunk straight and do not touch the wall. Hold this position for three seconds (Figure 4).

Kettlebell Windmill

**Figure 5:**
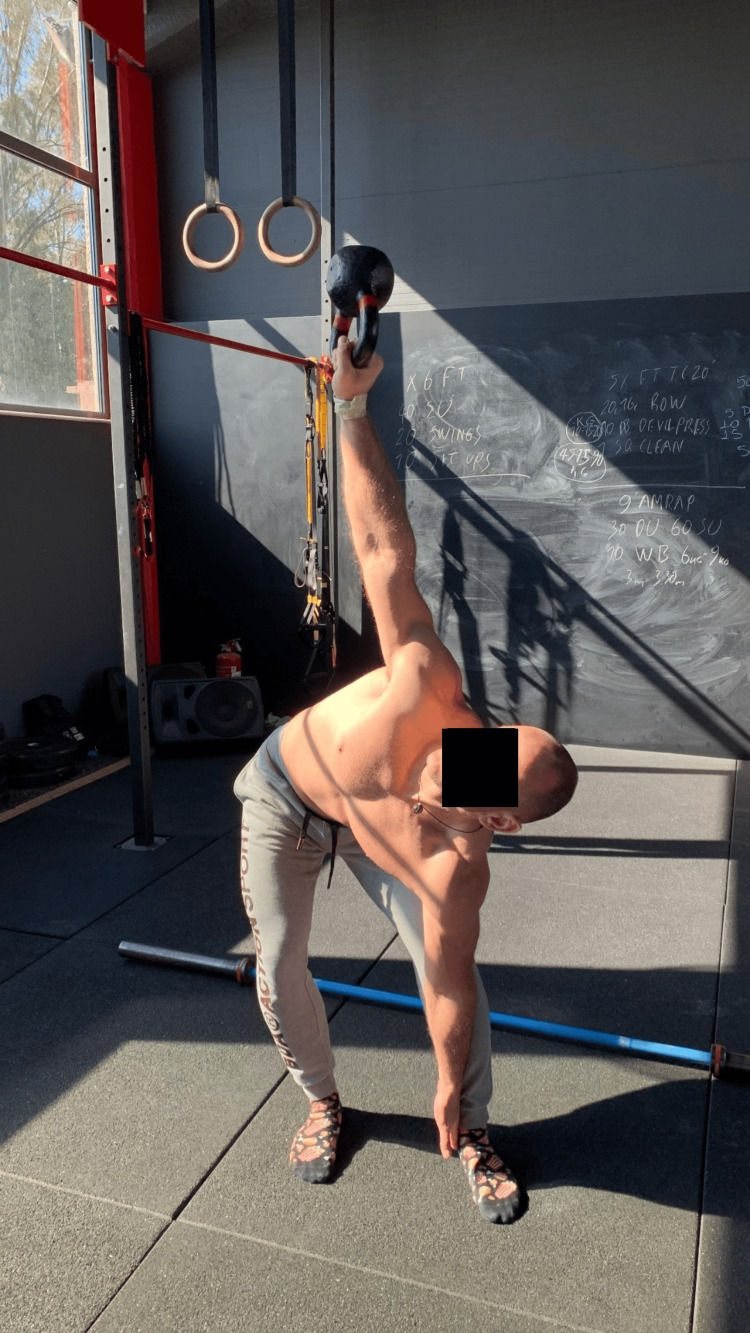
The windmill test.

Stand tall with your feet shoulder-width apart and toes pointing forward. Grasp the kb upside down with your right hand and place it overhead with your elbow straight. Now, turn both feet to the left until your big toe points 45° from the initial position. Place your left hand with the back side attached to your left leg and lower down to the ground as much as you can. Keep your chest open, your arm stable, and your elbow straight. Do not bend your knees more than unlocking. Return to the upright position and do it two more times. Do the same for the left hand. A one-minute time interval was provided between tests (Figure 5). (An overall qualitative score of the performed movement is registered for each hand.)

Sots Press

**Figure 6:**
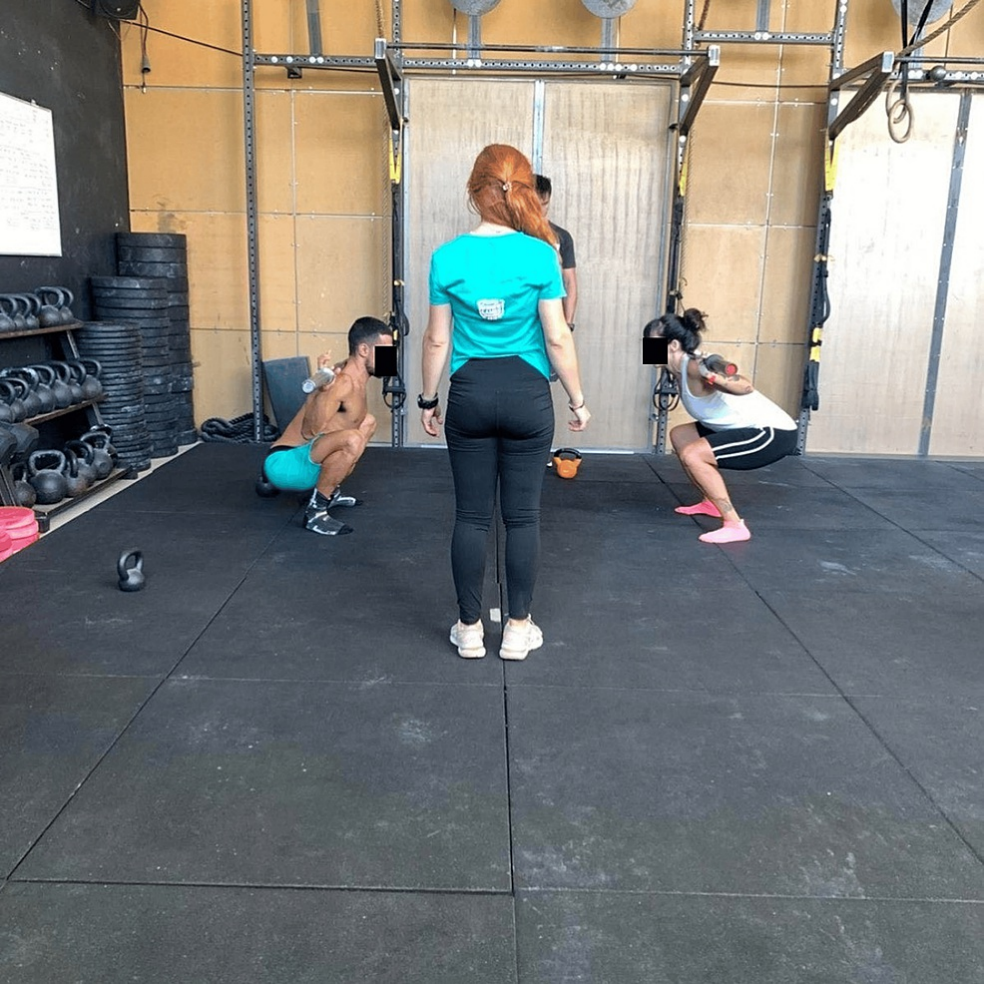
The Sots press test.

Stand tall with your feet slightly out of the shoulder width. Toes should point forward and slightly to the sides, about 15 degrees outward from the heel line. Grasp the bar at the same grip width as the stick before, using a snatch grip. Place it on a back rack position. Descend into a deep squat and maintain the initial grip and feet width. Now, press the bar three times with your body as much stable as you can. Do not raise your feet or ascend above the parallel. Hold the position for three seconds (Figure 6).

Each test gets scores from 0 to 3 on a four-point scale. A score of 0 is given when the movement causes the participant any kind of pain during the test, or the participant cannot perform the movement. A score of 1 is given when the participant performs the movement pattern using plenty of compensations. A score of 2 is given when a participant performs the movement successfully using minimal compensation. A score of 3 is given when a participant performs the movement correctly without using any compensation. The excellent score of CrossFit FABS is 18.

Data analysis

Data were analyzed using IBM SPSS Statistics for Windows, Version 28.0 (IBM Corp., Armonk, NY). Demographic data were analyzed for descriptive statistics. Nominal data were presented as frequency and percentage. Ordinal data were presented as mean values, minimum and maximum values, and standard deviation. To determine whether there were statistically significant correlations between scores given by the two examiners and between test and retest scores, the coefficient of Cohen's kappa and percent agreement were calculated for categorical variables [26,29]. For all statistical analyses, the significance level was set at the level of 0.05, based on the recorded *P*-value (*P* < 0.005).

## Results

Inter-rater reliability

Forty CrossFit athletes voluntarily participated in an inter-rater reliability study, comprising 26 men and 14 women aged between 20 and 50. The participants had an average age of 33.03 ± 7.67 years. Two independent raters conducted the examinations. The average weight was 75.39 ± 13.79 kg, and the average height was 1.74 ± 0.08 m. The body mass index (BMI) was calculated for each participant, and the average was 24.82 ± 3.26 kg/m^2^. The sample’s experience in CrossFit was between 1 and 120 months with a mean of 42.6 ± 31.16 months. The sample’s training volume was between 2 and 13 hours per week, with a mean of 5.01 ± 2.23 hours per week. Descriptive statistics for demographic data of the first subsample are presented in Table 1.

**Table 1:** Descriptive statistics for demographic data of the inter-rater reliability sample. BMI, body mass index

	Minimum	Maximum	Average	Standard deviation
Age (years)	20	50	33.03	7.67
Weight (kg)	47.5	105	75.39	13.79
Height (m)	1.59	1.90	1.74	0.08
BMI (kg/m^2^)	18.79	33.14	24.82	3.26
Training volume (hours/week)	4	2	5.01	2.23

The Cohen's kappa coefficient for the reliability of the measurements between the two examiners exceeded 0.8 in all tests. The air squat and OHS with the stick facing the wall exhibited the lowest values, with 0.824 and 0.890, respectively. These values indicate strong reliability in the measurements conducted. All the other tests showed almost excellent reliability, with kappa values of 0.965 for the shoulder mobility test, wall angel test, and windmill test for dominant upper extremity (UE); 0.966 for the shoulder mobility test for the nondominant UE; 0.929 for the windmill test for nondominant UE; and 1.0 for the Sots press test. Agreement among raters ranged from 90% to 100%. The correlations between values revealed statistically significant results, as evidenced by a P-value of 0.000 in all cases (Table 2).

**Table 2:** Cohen's kappa coefficient for inter-rater reliability. OHS, overhead squat; UE, upper extremity

Variables	*Κ*-value	Standard error	Statistically significant *P*-value	Percentage agreement among raters
Air squat	0.824	0.073	<0.001	90
Shoulder mobility for dominant UE	0.965	0.035	<0.001	97.5
Shoulder mobility for nondominant UE	0.966	0.033	<0.001	97.5
Wall angel	0.965	0.035	<0.001	97.5
OHS	0.890	0.061	<0.001	92.5
Windmill for dominant UE	0.965	0.034	<0.001	97.5
Windmill for nondominant UE	0.929	0.048	<0.001	95
Sots press	1.000	0.000	<0.001	100

Test-retest reliability

For the investigation of test-retest reliability, researchers enrolled 20 collegiate athletes participating in various sports. Ten men and 10 women, aged between 18 and 30 years, with a mean age of 21.25 ± 3.74 years, underwent evaluation twice with a one-week interval between assessments. The average weight of that sample was 67.03 ± 13.28 kg, and the average height was 1.71 ± 0.09 m. BMI was computed for each participant in the group, resulting in an average BMI of 22.8 ± 2.48 kg/m². Their average training volume was 9.2 ± 3.25 hours per week. The demographic characteristics of the test-retest study’s sample are presented in detail in Table 3.

**Table 3:** Descriptive statistics for demographic data of the test-retest reliability sample. BMI, body mass index

	Minimum	Maximum	Average	Standard deviation
Age (years)	18	30	21.25	3.74
Weight (kg)	51	94	67.03	13.28
Height (m)	1.57	1.87	1.71	0.09
BMI (kg/m^2^)	19.28	29.67	22.8	2.48
Training volume (hours/week)	4	17	9.2	3.25

The statistical analysis of the test-retest data yielded Cohen's kappa values ranging from 0.661 and higher for all variables of the functional assessment instrument. The shoulder mobility for the nondominant UE test showed strong reliability, and the Sots press test showed almost excellent reliability with *k*-values of 0.818 and 0.906, respectively. The intra-rater reliability for the remaining tests exhibited a moderate range, with kappa values of 0.661 for the windmill test for the dominant UE, 0.697 for the wall angel test, 0.703 for air squat, and 0.732 for OHS. Additionally, it showed moderate to strong reliability, with kappa values of 0.780 for the shoulder mobility test for the dominant UE and 0.776 for the windmill test for the nondominant UE. The percentage agreement between the two measurements ranged from 75% to 95%. The statistical significance of test-retest correlations among values was <0.001 for all the items of the battery (Table 4).

**Table 4:** Cohen’s kappa coefficient for test-retest reliability. OHS, overhead squat; UE, upper extremity

Variables	*k*-value	Standard error	Statistically significant *P-*value	Percentage test-retest agreement
Air squat	0.703	0.125	<0.001	80
Shoulder mobility for dominant UE	0.780	0.111	<0.001	85
Shoulder mobility for nondominant UE	0.818	0.122	<0.001	90
Wall angel	0.697	0.140	<0.001	80
OHS	0.732	0.131	<0.001	85
Windmill for dominant UE	0.661	0.129	<0.001	75
Windmill for nondominant UE	0.776	0.117	<0.001	85
Sots press	0.906	0.087	<0.001	95

## Discussion

This study showed that the reliability of the innovative battery of tests, CrossFit FABS, ranged from moderate to excellent for all included tests [26]. Following McHugh's interpretation of Cohen’s kappa, values below 0.60 indicated a lack of agreement among raters and suggested untrustworthy results, as approximately half of the data might be erroneous [29]. Statistical significance was observed to be lower than 0.001 for all tested correlations. The lowest kappa value among the outcomes, specifically 0.661 for the test-retest of the windmill on the dominant side, still exceeded the optimal threshold for characterizing the reliability of the test battery. CrossFit FABS was composed of six individual tests representing the essential movement patterns of CrossFit. The introduced functional assessment instrument aimed to reveal technical and coordination deficits that could lead to shoulder injuries during sports. For this purpose, the selected exercises were not limited to being sport-specific; they aimed to evaluate not only shoulder adequacy but also spine function and motor control [19,20]. The wall angel assessed UE flexibility, thoracic and cervical spine mobility in the sagittal plane, as well as movement coordination. The test-retest reliability of this exercise was investigated as part of a novel self-reported spine functional scale [28]. The calculation of the Spearman correlation coefficient (*r*) indicated fair reliability between repeated measurements over two weeks (*r* = 0.051, *P* < 0.01). Those results are in dissonance with the present study *k*-values, which showed moderate to strong correlation for the test-retest and inter-rater reliability, respectively. The exceptionally low values of the previous study [28] may be attributed to the self-reported assessment procedure, which was conducted by nonmedical professionals.

The novel instrument was designed to be sport-specific; therefore, fundamental exercises were selected, which were familiar to CrossFit athletes and used as preparation for basic movements or as accessories to improve performance. As it was structured to respond to various levels of CF athletes, from amateurs to elite, the difficulty of tests gradually increased. Simple functional tests such as shoulder mobility tests showed stronger correlations between measurements compared to more complicated tests such as squats. Air squats (*k *= 0.824, *P *< 0.001) and OHS (*k *= 0.890, *P* < 0.001) were the tests that demonstrated weaker correlations than the rest of the tests in the CrossFit athletes' group. Morgan et al. [30] reported similar findings regarding the inter-rater reliability of the updated version of the FMS. The intraclass correlation coefficient (ICC) was higher for shoulder mobility tests on both sides (ICC = 0.85 for the right and ICC = 0.94 for the left shoulder) compared to the deep squat test (ICC = 0.78), with a 95% confidence interval. The complexity of those movements and the wide variation of compensations that could be embraced were crucial factors for those results. However, the Sots press test demonstrated remarkably high correlations in both reliability sub-studies. This highly demanding exercise, known as the press-in-the-snatch, necessitates ankle, hip, trunk, and shoulder mobility, trunk stability strength, mid and upper back extension strength, upper body overhead strength, balance, accuracy, and comfort in the receiving position of a deep OHS. Therefore, it is hypothesized that the high-performance prerequisites of this movement infuse precision into the scoring process.

A discrepancy between the inter-rater and test-retest reliability results was observed. In contrast to the study by Popchak et al. [25], reliability among different raters was proved to be higher than the test-retest reliability. This could be attributed to the fact that in this study, both examiners evaluated the same motion pattern performed by each participant simultaneously. On the other hand, the test-retest assessment took place at different time points, which might influence the variability of those complex exercises. The results of the test-retest group displayed a wider variation than the inter-rater group. The reason could be that those athletes were not familiar with the specific exercises. However, performance did not appear to be influenced by the practice effect, as retest scores were not higher than test scores for the entire sample. In addition, the interpretation of lateral differences could be influenced by the biomechanical characteristics of the sports represented in the sample. The sample's sports did not involve symmetrical use of the UEs, a pattern that is more common among CrossFit athletes [23]. Four of the CrossFit FABS tests concerned bilateral movements, but the shoulder mobility and windmill tests evaluated upper limbs separately. This may be beneficial for demonstrating symmetry in extreme rotatory positions and the overhead position with stabilization demands, respectively [28].

Contrary to lower extremities, functional ability testing of the UEs in sports has not been studied extensively yet. Moreover, there is no evidence for reliable CrossFit-specific functional evaluation according to current published research. Consequently, the rest of the tests included in this study were novel, and further comparison with other studies is precarious.

Regarding the limitations of this study, first, a convenience sample was used for the test-retest reliability study. Volunteers were college students involved in sports other than CrossFit, and this might have influenced the results. However, the limited familiarization of the participants with the exercises possibly underestimated the results. We had expected the results to be even better for CrossFit athletes who are familiar with these routines. The inter-examiner reliability was tested in a second phase, after ensuring that the more specific and difficult-to-access sample of CrossFit athletes and the involvement of a second investigator posed negligible risks. Additionally, there was a disproportion in sample sizes between the two reliability evaluations. The test-retest study had fewer participants than the inter-rater reliability study, which made the comparison of the results questionable. Further research is suggested to investigate the validity and predictive ability of our proposed test battery for shoulder joint injuries in CrossFit athletes.

## Conclusions

This study is unique in that the authors created and used a sport-specific tool to assess the functional ability of CrossFit participants. The shoulder joint proved to be the most prevalent injured area of the body among those athletes. Insufficient kinetic control, muscular strength asymmetries, inadequate core stability, and limited flexibility are some modifiable factors associated with injury incidence. Applied evaluation methods such as dynamometry and goniometry are well-documented as valid indicators of injury risk in sports; on the contrary, a lack of reliable functional performance tests related to UE injuries is observed. The present functional test-battery development aimed to reveal deficits that can potentially lead to shoulder injury incidents. The results of this study confirm the reliability of the innovative instrument, suggesting its use in the individual functional assessment of CrossFit participants or as part of a screening, including optimal evaluation of general mobility, strength, and endurance.
